# Obtention of solar cell parameters, through convergence of iterative cycles. Part 2: Application to experimental current-voltage measurements

**DOI:** 10.1016/j.heliyon.2022.e10548

**Published:** 2022-09-09

**Authors:** Victor-Tapio Rangel-Kuoppa

**Affiliations:** Department of Physics, Lancaster University, Lancaster, United Kingdom

**Keywords:** Solar cell parameter extraction, Shunt resistance, Series resistance, Ideality factor, Light current, Saturation current

## Abstract

In this Part 2 of this series of articles, the application of the iterative cycles CycleA and CycleB proposed in Part 1, to determine the solar cell parameters (the shunt resistance ($R_{sh}$), the series resistance ($R_{s}$), the ideality factor (*n*), the light current ($I_{\mathrm{lig}}$), and the saturation current ($I_{\mathrm{sat}}$)) on experimental current voltage (*IV*) and current density (*JV*) curves, is given. Several number of measured points per voltage ($P_{V}$) are attempted, from approximately $P_{V}=1\;\frac{measured\;points}{V}$ to $P_{V}=52\;\frac{measured\;points}{V}$. In one case, the application of the iterative cycles to *IV* curves showing the roll-over effect is discussed, while in another case, their application to solar panels is analysed, revealing that the iterative cycles can also be used in the case of solar panels, and not only for laboratory-made solar cells, in voltage ranges larger than [0 V,1 V]. Also, cases in darkness and under illumination are evaluated. In most cases, reasonable values are obtained for $R_{sh}$, $R_{s}$, $n,I_{\mathrm{sat}}$ and $I_{\mathrm{lig}}$, which simulated properly the *IV* or *JV* curves.

## 1 Introduction

Humanity is well aware of the ecological problems it is facing, due to climate change triggered by fossil fuels gases emitted to the atmosphere, during the last centuries. At the same time, it is forecasted that the energy consumption shall reach 30 TW by the year 2050, increasing from its current value of 10 TW. These two facts encourage humanity to reduce its CO_2_ and other carbon related gases emission, while increasing the energy production. Solar panels and solar cells have shown to be suitable candidates to accomplish these two goals, as they yield cheap energy in a nature-friendly way [1].

Due to its simplicity, the one-diode solar cell model is the most used model to explain solar cells, (see equation (1) and Fig. 1 in [2]). In this model, the parameters are the shunt resistance ($R_{sh}$), the series resistance ($R_{s}$), the light current ($I_{\mathrm{lig}}$), the ideality factor (*n*), and the saturation current ($I_{\mathrm{sat}}$), according to Fig. 1 in [2]. It is the current voltage (*IV*) measurement the one most widely used to obtain these five solar cell parameters, both under illumination and in darkness [3], [4].

A brief description of the importance and physical information provided by $R_{sh}$, $R_{s}$, $I_{\mathrm{lig}}$, $I_{\mathrm{sat}}$, and *n* can be found in the Introduction in Part 1 of this series of articles [5]. Also there, a summary of the available techniques to obtain them is described. Briefly, a myriad of methods can be found in the literature, based in Monte Carlo simulations, artificial neuronal networks, non-linear least-squares method, exponential model, or ab initio calculations [6], [7], [8], [9], [10], [11], [12], [13], [14], [15]. It is worth mentioning here the study by Khan et al., where they reviewed the limitations of these main techniques [16].

This explains the intention of this Part 2 of this series of articles: to show the suitability of the iterative cycles proposed in Part 1 [5], to obtain the five solar cell parameters, within the one-diode model, for different types of solar cells and solar panels, both under illumination and in darkness.

In this Part 2 of this series of articles, the iterative cycles CycleA and Cycle B, proposed in [5], are applied to experimental current (*I*) *vs.* voltage (*V*) (*IV*) and current density (*J*) *vs. V* (*JV*) curves reported in the literature, to properly obtain the solar cell parameters (the shunt resistance ($R_{sh}$), the series resistance ($R_{s}$), the ideality factor (*n*), the light current ($I_{\mathrm{lig}}$), and the saturation current ($I_{\mathrm{sat}}$). Briefly, these cycles consist on the following: first, the Cheung method, which was originally proposed for Schottky contacts [17], [18], [19], is extended to the solar cell equation, within the one diode solar cell model (equation 1 in [2]), yielding *n* and $R_{s}$. Next, Procedure A and B proposed in [2], [20] are used to obtain $R_{sh}$ and $I_{\mathrm{sat}}$. Finally, a correction to $I_{\mathrm{lig}}$ is obtained (equation 15 in [5]), and the iterative cycle continues till some convergence criteria have been reached. Further details can be found in [5].

The same *IV* and *JV* curves discussed in [21], whose solar cell parameters extraction were examined according to the discussion in [22], are analysed in this article. Then, for simplicity purpose for the reader, this article is divided in the same sections as in [21]. Section 1, Introduction, is followed by Section 2, where the iterative cycles CycleA and CycleB are used in *IV* with a density of points $P_{V}<30\;\frac{measured\;points}{V}$, reported in the literature. In Section 3 a similar analysis is done, but in this case to *JV* measurements done with values of $P_{V}$ between $30\;\frac{measured\;points}{V}$ and $50\;\frac{measured\;points}{V}$. Section 4 follows, where *IV* and *JV* measurements done with $P_{V}>50\;\frac{measured\;points}{V}$ are analysed. Discussion of the results is given in each section and finally conclusions are given in Section 5.

## 2 *IV* measurements done with $P_{V}<30\;\frac{measured\;points}{V}$

In their study, Amiry et al. reported four *IV* curves (Fig. 7.a) in [23]) measured at temperatures of 30 °C, 35 °C, 43 °C and 49 °C, and an illumination power of 1030 W/cm^2^, of photovoltaic modules, using their built low-cost acquisition setup [23]. For clarity purposes for the reader, the same labelling used in [23] is used in this article. Amiry et al. mentioned they used 34 measurement points in their Fig. 7.a) in [23], and as it was done from 0 V to around 21 V, then their $P_{V}=1.6\;\frac{measured\;points}{V}$.

Amiry et al. stated they deduced their solar cell parameters using the Ortiz-Conde et al. [24] method and they reported them in their Table 2 in [23]. However, as it is discussed in Section 2 in [21], their deduced solar cell parameters do not reproduce their measured *IV* curves. CycleA and CycleB were used in this Section to obtain the proper solar cell parameters.

In the case of CycleA, the program CycleAmanual described in [5] was used. It was found that it always yielded unrealistic negative values for $R_{sh}$, independently of the number of cycles that were tried (not shown here). This is in agreement with the results exposed in [5]: for values of $P_{V}\leq 11\;\frac{measured\;points}{V}$, CycleA yields unrealistic negative $R_{sh}$.

**Figure 1:**
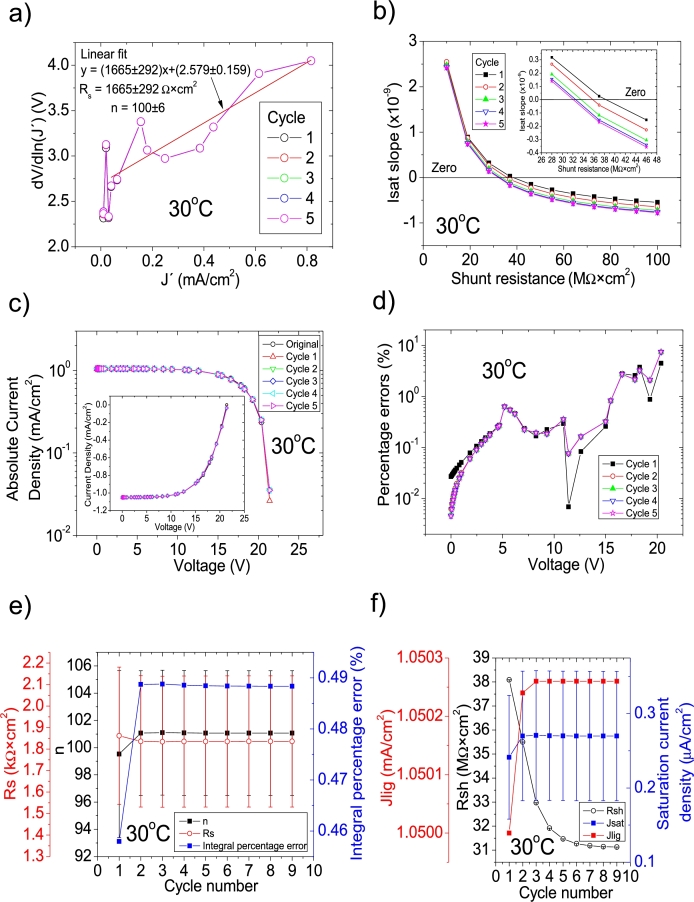
Nine cycles application of program CycleB to the *JV* curve shown in Fig. 1 in [21] for the 30 °C curve and the respective cycles steps shown as (a) linear fit of $\frac{\partial V}{\partial lnJ^{\prime }}=nkT+R_{s}\left(J^{\prime }\right)$ vs. *J*′, for the first five cycles, (b) plot of *m*_*sat*_*vs. R*_*sh*_ to obtain a root for *R*_*sh*_, for the first five cycles. (c) Logarithm plot of absolute *J* vs *V* of the original *JV* curve (in black) and for each resimulations done with the deduced solar cell parameters for the first five cycles. The same data are plot in the inset as *JV*. (d) Percentage errors between the original *JV* curve and each resimulation shown in (c). (e) Deduced *R*_*s*_ (red), *n* (black), and integral percentage errors (blue) for each cycle. (f) Deduced *R*_*sh*_ (black), *J*_*lig*_ (red), and *J*_*sat*_ (blue) for each cycle.

**Figure 2:**
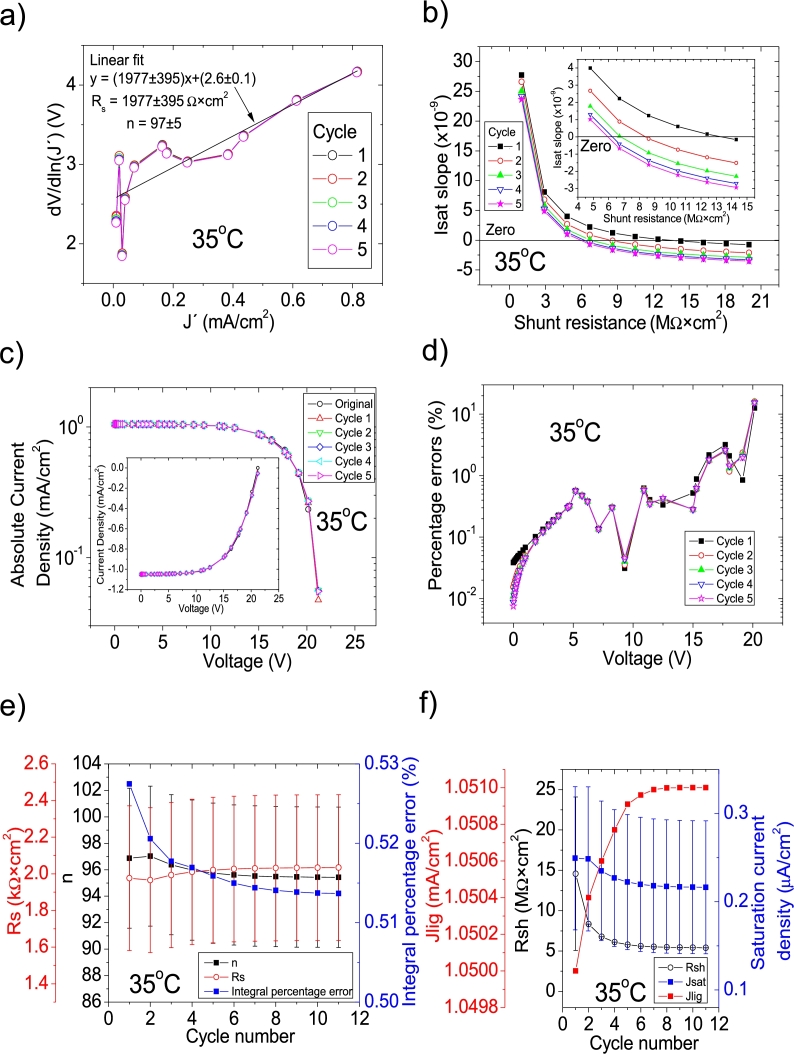
Eleven cycles application of program CycleB to the *JV* curve shown in Fig. 1 in [21] for the 35 °C curve and the respective cycles step shown as (a) linear fit of $\frac{\partial V}{\partial lnJ^{\prime }}=nkT+R_{s}\left(J^{\prime }\right)$ vs. *J*′, for the first five cycles, (b) plot of *m*_*sat*_*vs. R*_*sh*_ to obtain a root for *R*_*sh*_, for the first five cycles. (c) Logarithm plot of absolute *J* vs *V* of the original *JV* curve (in black) and for each resimulations done with the deduced solar cell parameters for the first five cycles. The same data are plot in the inset as *JV*. (d) Percentage errors between the original *JV* curve and each resimulation shown in (c). (e) Deduced *R*_*s*_ (red), *n* (black) and integral percentage errors (blue) for each cycle. (f) Deduced *R*_*sh*_ (black), *J*_*lig*_ (red), and *J*_*sat*_ (blue) for each cycle.

**Figure 3:**
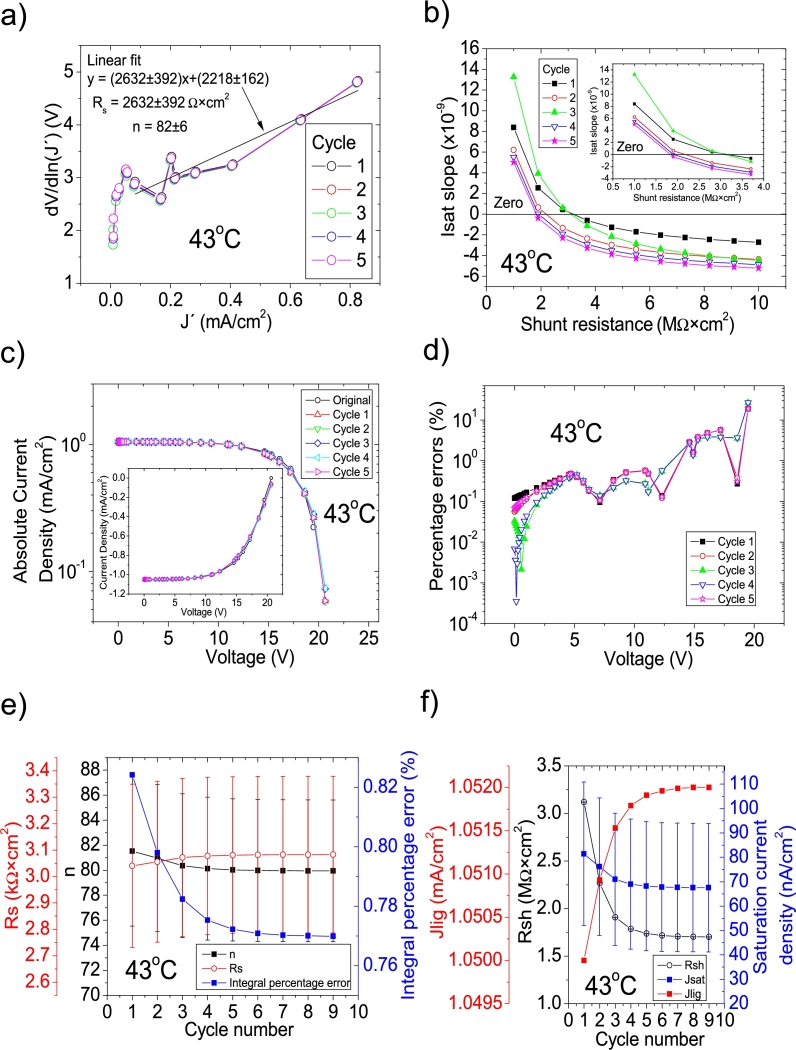
Nine cycles application of program CycleB to the *JV* curve shown in Fig. 1 in [21] for the 43 °C curve and the respective cycles steps shown as (a) linear fit of $\frac{\partial V}{\partial lnJ^{\prime }}=nkT+R_{s}\left(J^{\prime }\right)$ vs. *J*′, for the first five cycles, (b) plot of *m*_*sat*_*vs. R*_*sh*_ to obtain a root for *R*_*sh*_, for the first five cycles. (c) Logarithm plot of absolute *J* vs *V* of the original *JV* curve (in black) and for each resimulations done with the deduced solar cell parameters for the first five cycles. The same data are plot in the inset as *JV*. (d) Percentage errors between the original *JV* curve and each resimulation shown in (c). (e) Deduced *R*_*s*_ (red), *n* (black), and integral percentage errors (blue) for each cycle. (f) Deduced *R*_*sh*_ (black), *J*_*lig*_ (red), and *J*_*sat*_ (blue) for each cycle.

**Figure 4:**
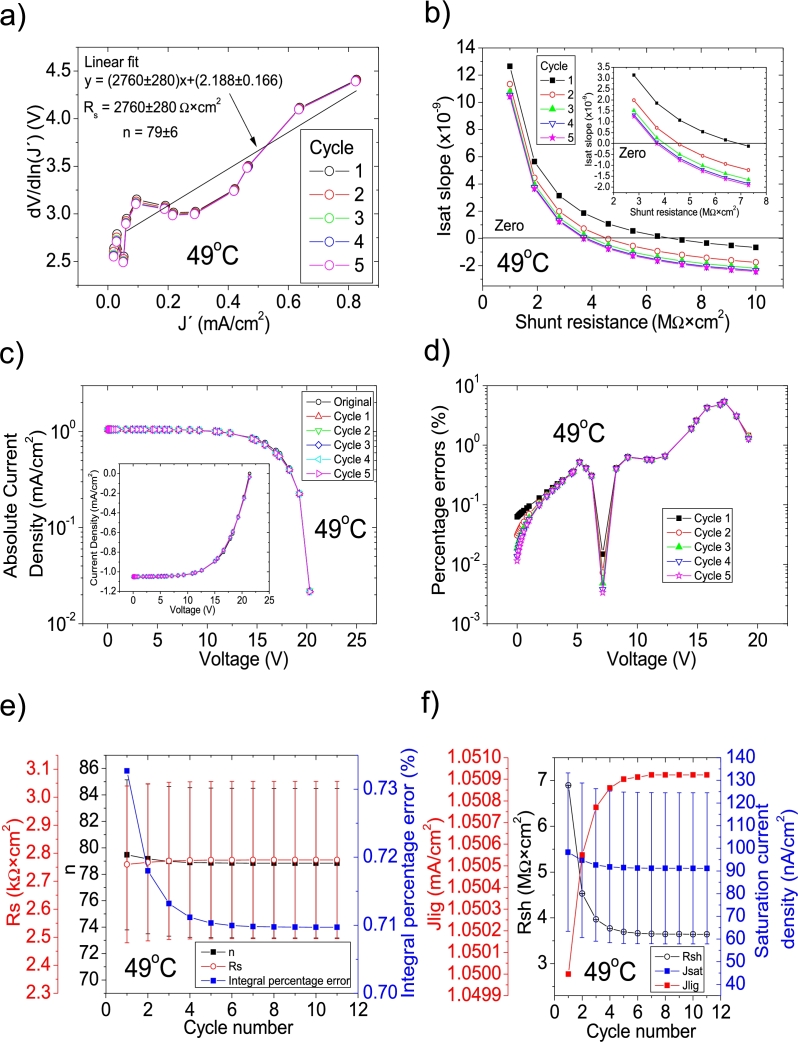
Eleven cycles application of program CycleB to the *JV* curve shown in Fig. 1 in [21] for the 30 °C curve and the respective cycles steps shown as (a) linear fit of $\frac{\partial V}{\partial lnJ^{\prime }}=nkT+R_{s}\left(J^{\prime }\right)$ vs. *J*′, for the first five cycles, (b) plot of *m*_*sat*_*vs. R*_*sh*_ to obtain a root for *R*_*sh*_, for the first five cycles. (c) Logarithm plot of absolute *J* vs *V* of the original *JV* curve (in black) and for each resimulations done with the deduced solar cell parameters for the first five cycles. The same data are plot in the inset as *JV*. (d) Percentage errors between the original *JV* curve and each resimulation shown in (c). (e) Deduced *R*_*s*_ (red), *n* (black), and integral percentage errors (blue) for each cycle. (f) Deduced *R*_*sh*_ (black), *J*_*lig*_ (red), and *J*_*sat*_ (blue) for each cycle.

CycleB was applied on the *JV* curves reported in Fig. 1 in [21], using the program CycleB commented in [5]. Briefly, this *JV* curves are the *I* value shown in Fig. 7.a) in [23] divided by their area of 449.28 cm^2^, reported in Table 1 in [23]. Further discussion on this can be found in Section 2 in [21]. The application of CycleB is shown in Fig. Figure 1, Figure 2, Figure 3, Figure 4. As can be seen in Fig. 1.abcdef)-4.abcdef), the extracted solar cell parameters derived here, reproduced well the *JV* curves, as the percentage errors are in general below 1%, increasing for voltages larger than 15 V to around 10%, when the voltage reaches 21 V. This is expectable, as *J* converges to zero as *V* approaches 21 V, increasing the percentage error. In the case of *JV* curves 30 °C, and 43 °C, nine cycles were necessary to obtain convergence in all the solar cell parameters, while eleven cycles were needed in the case of 35 °C and 49 °C.

Results of the application of the iterative CycleB in Fig. 1.abcdef)- 4.abcdef) are summarized in Table 1, together with the results exposed in Table 2 in [23] and in Table 1 in [21].

**Table 1:** Solar cell parameters for the solar cells studied in [23]. The superscript ^a^ refers to the values reported in Table 2 in [23], while the superscript ^b^ are the values obtained using the Ortiz-Conde et al. method in the *JV* curves of Fig. 1 in [21] and reported in Table 1 in [21]. The superscript ^c^ are the values deduced in this article, after convergence using CycleB.

Measurement	*R*_*sh*_ (Ω × cm^2^)	*R*_*s*_ (Ω × cm^2^)	*n*	$J_{\text{lig}}$ (A/cm^2^)	*J*_*sat*_ (A/cm^2^)
	^a^ 901	^a^ 1.5	^a^ 2.41	^a^ 3.76 × 10^−2^	^a^ 2.71 × 10^−6^
30 °C	^b^ (6 ± 3) × 10^5^	^b^ (1 ± 1) × 10^3^	^b^ 23 ± 62	^b^ (1.055 ± 0.003) × 10^−3^	^b^ 1 × 10^−9^
	^c^ (3.113 ± 0.00005) × 10^7^	^c^ (18 ± 3) × 10^2^	^c^ 101 ± 5	^c^ (1.05) × 10^−3^	^c^ (27 ± 9) × 10^−8^

	^a^ 862	^a^ 1.54	^a^ 2.36	^a^ 3.77 × 10^−2^	^a^ 2.83 × 10^−6^
35 °C	^b^ (8 ± 5) × 10^5^	^b^ (1 ± 1) × 10^3^	^b^ 48 ± 57	^b^ (1.054 ± 0.004) × 10^−3^	^b^ 1 × 10^−9^
	^c^ (5.4107 ± 0.0005) × 10^6^	^c^ (20 ± 4) × 10^2^	^c^ 95 ± 5	^c^ (1.051) × 10^−3^	^c^ (22 ± 8) × 10^−8^

	^a^870	^a^1.59	^a^2.32	^a^3.77 × 10^−2^	^a^4.13 × 10^−6^
43 °C	^b^ (6 ± 3) × 10^5^	^b^ (1 ± 1) × 10^3^	^b^ 22 ± 59	^b^ (1.055 ± 0.003) × 10^−3^	^b^ 1 × 10^−9^
	^c^ (1.7017 ± 0.0005) × 10^6^	^c^ (30 ± 3) × 10^3^	^c^ 80 ± 6	^c^ (1.052) × 10^−3^	^c^ (7 ± 3) × 10^−8^

	^a^553	^a^1.64	^a^2.28	^a^3.77 × 10^−2^	^a^4.69 × 10^−6^
49 °C	^b^ (6 ± 3) × 10^5^	^b^ (1 ± 1) × 10^3^	^b^ 22 ± 58	^b^ (1.055 ± 0.003) × 10^−3^	^b^ 1 × 10^−9^
	^c^ (3.6423 ± 0.0005) × 10^6^	^c^ (28 ± 3) × 10^3^	^c^ 79 ± 6	^c^ (1.05) × 10^−3^	^c^ (7 ± 3) × 10^−8^

As can be seen from Table 1, the results reported by Amiry et al. for $R_{sh}$ are four orders of magnitude lower than the correct values, while their $R_{s}$ values are three orders lower than the correct values. On the other hand, $J_{\mathrm{sat}}$ are two orders of magnitude higher than the correct values. Finally, *n* ($J_{\mathrm{lig}}$) are around one order of magnitude lower (higher) than the correct values. Nevertheless, all the solar cell parameters show the same tendency as the one exposed by Amiry et al. in their Fig. 10 in [23], namely, $J_{\mathrm{sat}}$ and $R_{s}$ (*n* and $R_{sh}$) increase (decrease) as temperature increases.

It is worth noticing that $R_{sh}$, $R_{s}$ and *n* extracted using the Ortiz-Conde et al. [24] method in Section 2 in [21] (superscript ^b^ in Table 1) are between the values reported by Amiry et al. [23] and the values obtained in this article, while $J_{\mathrm{sat}}$ extracted in this study is between one and two orders of magnitude larger. This reveals that CycleB is more suitable technique to obtain the solar cell parameters, in a case where $P_{V}$ is below $2\;\frac{measured\;points}{V}$, provided the voltage range is around [0 V, 21 V].

The conclusions exposed in this Section, show that the results exposed by Amiry et al. in their Table 2-5 in [23] should be revised, including those deduced using their iterative method in the case of the single diode model, or their conventional and alternative methods for the case of the double diode model, as they are very similar to those obtained using the Ortiz-Conde et al. method shown in their Table 2 in [23].

**Figure 5:**
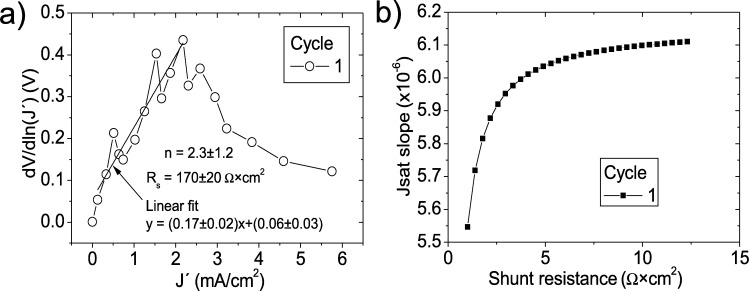
(a) Linear fit of $\frac{\partial V}{\partial lnJ^{\prime }}=nkT+R_{s}\left(J^{\prime }\right)$ vs. *J*′, in [0 mA/cm^2^, 2 mA/cm^2^], (b) plot of *m*_*sat*_*vs. R*_*sh*_ to obtain a root for *R*_*sh*_, for the first cycle (it is always positive).

When Ortiz-Conde et al. proposed their graceful idea to use the Co-content function CC(V,I) (or CC(V,J)) to obtain the solar cell parameters, they applied it to the *JV* curve measured in a plastic solar cell, with a $P_{V}=26.56\;\frac{measured\;points}{V}$, in the [0 V, 0.753 V], *i.e.*, 20 measurement points in total [24]. Their deduced solar cell parameters reported in the inset of Fig. 2 of [24] properly reproduce the *JV* curve (see Fig. 5.a) in [21]). They mentioned they used a fourth-order Simpson-type numerical integration to calculate their CC(V,J) [24]. Unfortunately, a number of integration points of the form 4u+1, where *u* is a natural number, are necessary to apply this numerical integration method, i.e., their 20 number points in Fig. 2 in [24] did not satisfy this criterion (see discussion in Section 2 in [21]), and then it was not possible to recalculate CC(V,J)) using this integration method in [21]. Thus, the usual trapezoidal integration method was used in [21]. As it is discussed in Section 2 in [21], using this trapezoidal method, only $J_{lig}$ is similar to the value of $J_{lig}=7.94\;\frac{\text{mA}}{{\text{cm}}^{2}}$ reported by Ortiz-Conde et al. [24], and $R_{sh}=340\pm 30\;\Omega \times {\text{cm}}^{2}$ is in the same order than their reported magnitude of $R_{sh}=G_{P}^{-1}=197.23\;\Omega \times {\text{cm}}^{2}$ [24]. Regarding $R_{s}$ and *n*, they were unrealistically negative. CycleA and CycleB (using program CycleAmanual and CycleB described in [5]) were used in an attempt to confirm the solar cell parameters reported by Ortiz-Conde et al., namely $R_{s}=8.59\;\Omega \times {\text{cm}}^{2}$, n=2.31, $J_{sat}=13.6\;\frac{\text{nA}}{{\text{cm}}^{2}}$, $J_{lig}=7.94\;\frac{\text{mA}}{{\text{cm}}^{2}}$ and $R_{sh}=G_{P}^{-1}=197.23\;\Omega \times {\text{cm}}^{2}$.

When doing the linear fit of $\frac{\partial V}{\partial lnJ^{\prime }}$ *vs.* $J^{\prime }$ during the first cycle, for both CycleA and CycleB, the curve shows a change to a negative slope around $J^{\prime }=2\text{ mA}$ (see Fig. 5.a)). This seems to be related to the fact that in this case $\frac{R_{s}}{R_{sh}}=\frac{8.59\;\Omega \times {\text{cm}}^{2}}{197.23\;\Omega \times {\text{cm}}^{2}}=0.043$, forty times larger than the $\frac{R_{s}}{R_{sh}}=0.001$ used in [5] and also the $\frac{R_{s}}{R_{sh}}$ values appearing in the other cases examined in this article. This value of $\frac{R_{s}}{R_{sh}}=0.043$ causes that the term $\frac{V-JR_{s}}{R_{sh}}$ is nonnegligible for large values of *J* in the first cycle, causing the plot to change slope. This shows that applicability of CycleA and CycleB is limited by a condition on $\frac{R_{s}}{R_{sh}}$. This is currently being investigated and will be reported elsewhere.

Nevertheless, if the linear fitting is done on the range [0.2 mA, 2 mA], values of $R_{s}=170\pm 20\;\Omega \times {\text{cm}}^{2}$ and n=2.3±1.2 are obtained (see Fig. 5.a)). This value of *n* is in reasonable agreement with the value of n=2.31 reported by Ortiz-Conde et al., however $R_{s}$ is two orders of magnitude larger than the value of $R_{s}=8.59\;\Omega \times {\text{cm}}^{2}$ reported by Ortiz-Conde et al. This value of $\frac{R_{s}}{R_{sh}}$ causes, that when Procedure B was used next in CycleB, it was never possible to find a root in the curve of $m_{sat}$ *vs.* $R_{sh}$, being always positive and increasing, despite any $R_{sh}$ range value attempted (see as an example Fig. 5.b)), while using Procedure A in CycleA, $R_{sh}$ was found to be always unrealistically negative (not shown here). Then, it was impossible to use CycleA and CycleB to obtain the solar cell parameters, revealing that a condition $\frac{R_{s}}{R_{sh}}$ is necessary for their suitable application, which is currently being investigated.

## 3 IV measurements done with $P_{V}>30\frac{measured\;points}{V}$ and $P_{V}<50\frac{measured\;points}{V}$

In their article, Rejón et al. [25] claimed they used the Ortiz-Conde et al. [24] method to determine the solar cell parameters of their oxygen-CHClF_2_ activated CdS/CdTe solar cells, samples labelled A, B and C in [25], both in darkness and under illumination. These same values are exposed in Table I and II in [26]. Only $R_{sh}=6\times 10^{6}\;\Omega \times {\text{cm}}^{2}$ in Table I in [26] differs from $R_{sh}=6\times 10^{4}\;\Omega \times {\text{cm}}^{2}$ in Table 2 in [25], for the C sample in [25] and ArOF in [26], respectively. Comparison of the samples names suggests samples A, B and C in [25] are samples Ar, ArF and ArOF in [26]. In [27] similar samples are reported, with an additional one, named N-O-Freon, included. Their *JV* curves are reported in darkness (Fig. 1 in [27]) and under illumination (Fig. 2 in [27]). Further discussion of this can be found in Section 3 in [21].

For coherence purpose for readers, the same sample labelling and symbols are used in this article as in [27], while the *JV* curves can be seen in Fig. 8.ab) in [21], together with simulations done using the solar cell parameters reported in [25], [26], [27].

For clarity purposes, this Section is divided in two subsections, namely Subsection 3.1, where the analysis of their reported *JV* curves, in darkness (Fig. 8.a) in [21]) is done, while in Subsection 3.2 the analysis of their reported *JV* curves under illumination (Fig. 8.b) in [21]) is given. Discussion of both cases is given at the end of this Section.

### 3.1 Measurements in darkness

As it is discussed in Section 3 in [21], the solar cell parameters do not reproduce at all the *JV* curves for the Ar-Freon sample, revealing something is wrong with them, while they reasonably reproduce the *JV* measurements in the case of samples Ar and N-O-Freon, and also for the case Ar-O-Freon, if the value of $R_{sh}=6\times 10^{6}\;\Omega \times {\text{cm}}^{2}$ is used (see Fig. 8.a) in [21]). The Ortiz-Conde et al. method [24] was used on them in [21] to reproduce the results, and only in the case of the Ar-O-Freon sample, $R_{sh}$, $R_{s}$, *n* and $I_{\mathrm{lig}}$ were all positive, and partially similar to those reported in Table II in [27] (see Table 4 and discussion in Section 3 in [21]). When $I_{\mathrm{sat}}$ was deduced following the Ortiz-Conde et al. method [24], completely different values to those reported in Table I and II in [26] and Table II in [27] were obtained (see Fig. 11 and discussion in Section 3 in [21]). Then it is unclear how the solar cell parameters were so accurately deduced for Ar, N-O-Freon, and Ar-O-Freon samples, as it was impossible to deduce them so accurately using the Ortiz-Conde et al. method, as it was proven in [21]. The accurate determination in these cases suggests that the solar cell parameters were known beforehand, i.e., the *JV* curves were manufactured.

**Figure 6:**
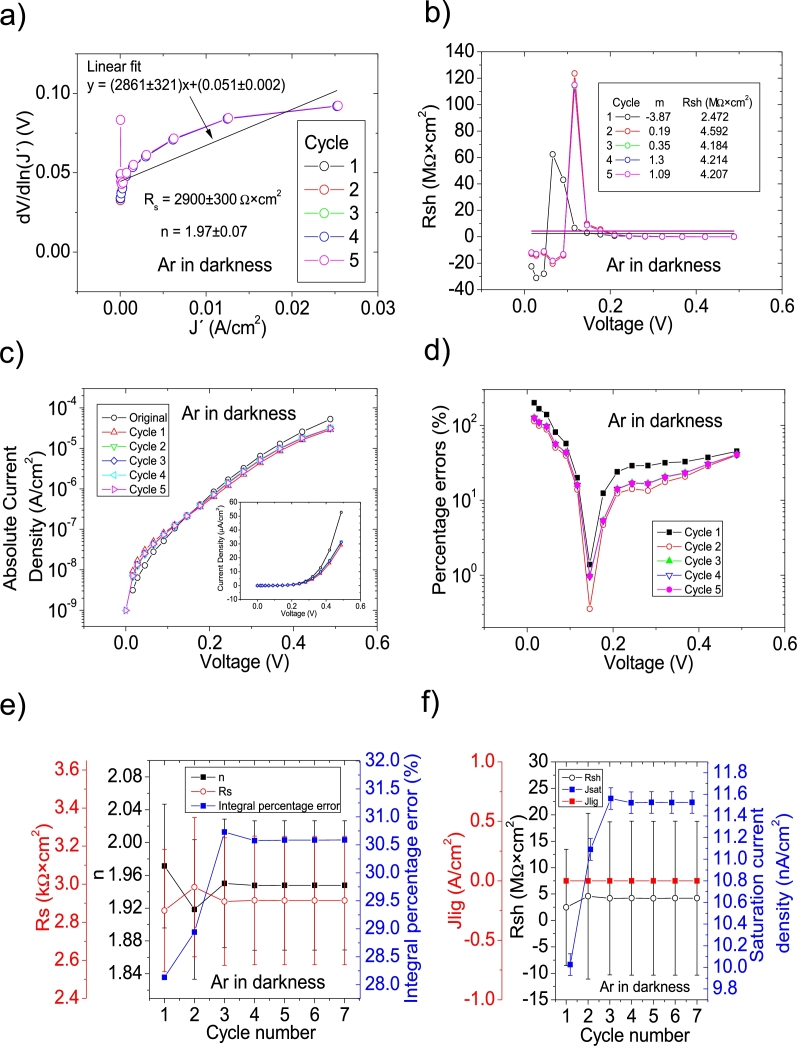
Seven cycles application of program CycleAmanual to the *JV* curve shown in Fig. 8.a) in [21] for the Ar curve in darkness, and the respective cycles steps shown as (a) linear fit of $\frac{\partial V}{\partial lnJ^{\prime }}=nkT+R_{s}\left(J^{\prime }\right)$ vs. *J*′, for the first five cycles, (b) plot of *R*_*sh*_*vs. V* varying *I*_*sat*_ for each cycle to obtain a horizontal linear fit. The horizontal black, red, green, blue and magenta lines are the linear fitting for Cycles 1 to 5, respectively. The value m is the slope of each linear fit, while the *R*_*sh*_ in the table is the obtained constant of the linear fitting. (c) Logarithm plot of absolute *J* vs *V* of the original *JV* curve (in black) and for each resimulations done with the deduced solar cell parameters for the first five cycles. The same data are plot in the inset as *JV*. (d) Percentage errors between the original *JV* curve and each resimulation shown in (c). (e) Deduced *R*_*s*_ (red), *n* (black) and integral percentage errors (blue) for each cycle. For clarity purposes, the error bars were removed. (f) Deduced *R*_*sh*_ (black), *J*_*lig*_ (red) and *J*_*sat*_ (blue) for each cycle.

**Figure 7:**
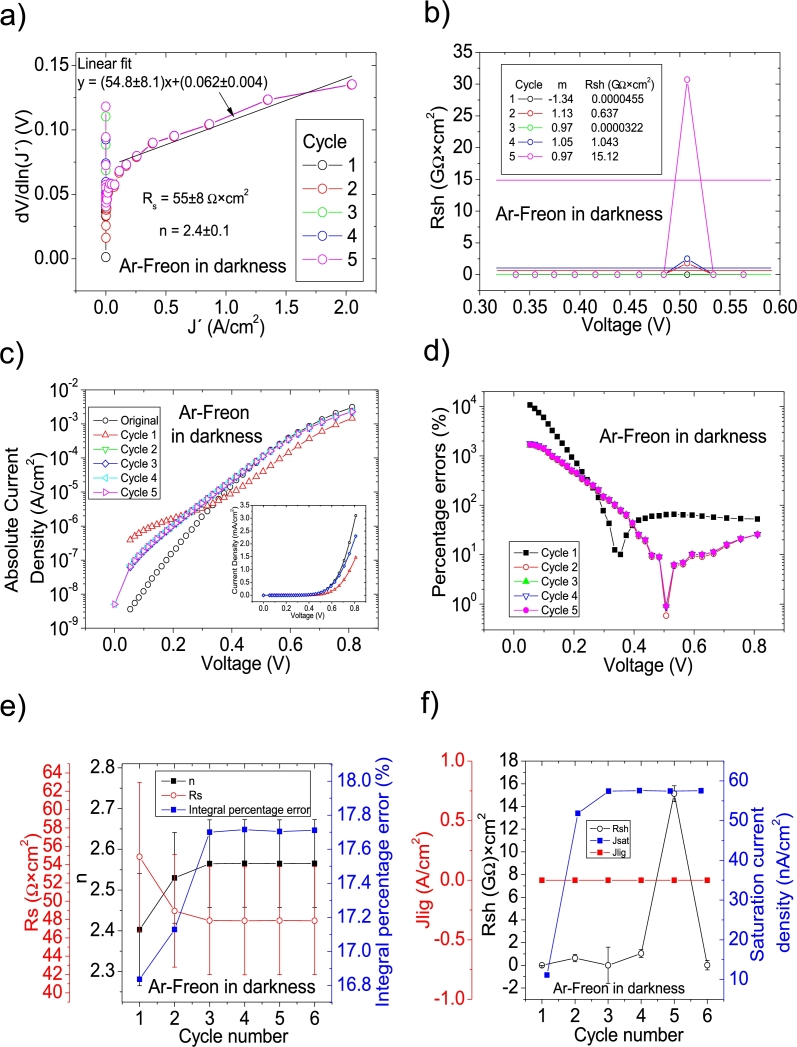
Seven cycles application of program CycleAmanual to the *JV* curve shown in Fig. 8.a) in [21] for the Ar-Freon curve in darkness, and the respective cycles steps shown as a) linear fit of $\frac{\partial V}{\partial lnJ^{\prime }}=nkT+R_{s}\left(J^{\prime }\right)$ vs. *J*′, for the first five cycles, (b) plot of *R*_*sh*_*vs. V* varying *I*_*sat*_ for each cycle to obtain a horizontal linear fit. The horizontal black, red, green, blue and magenta lines are the linear fitting for Cycles 1 to 5, respectively. The value m is the slope of each linear fit, while the *R*_*sh*_ in the table is the obtained constant of the linear fitting. (c) Logarithm plot of absolute *J* vs *V* of the original *JV* curve (in black) and for each resimulations done with the deduced solar cell parameters for the first five cycles. The same data are plot in the inset as *JV*. (d) Percentage errors between the original *JV* curve and each resimulation shown in (c). (e) Deduced *R*_*s*_ (red), *n* (black) and integral percentage errors (blue) for each cycle. For clarity purposes, the error bars were removed. (f) Deduced *R*_*sh*_ (black), *J*_*lig*_ (red) and *J*_*sat*_ (blue) for each cycle.

**Figure 8:**
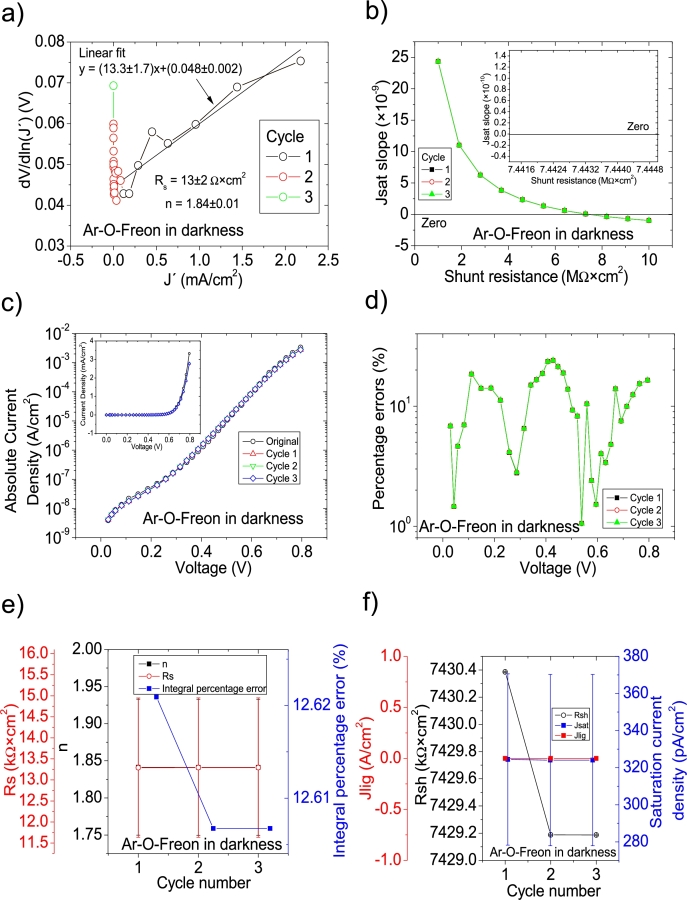
Three cycles application of program CycleB to the *JV* curve shown in Fig. 8.a) in [21] for the Ar-O-Freon curve in darkness, and the respective cycles steps shown as (a) linear fit of $\frac{\partial V}{\partial lnJ^{\prime }}=nkT+R_{s}\left(J^{\prime }\right)$ vs. *J*′, (b) plot of *m*_*sat*_*vs. R*_*sh*_ to obtain a root for *R*_*sh*_. (c) Logarithm plot of absolute *J* vs *V* of the original *JV* curve (in black) and for each resimulations done with the deduced solar cell. The same data are plot in the inset as *JV*. (d) Percentage errors between the original *JV* curve and each resimulation shown in (c). (e) Deduced *R*_*s*_ (red), *n* (black) and integral percentage errors (blue) for each cycle. For clarity purposes, the error bars were removed. (f) Deduced *R*_*sh*_ (black), *J*_*lig*_ (red) and *J*_*sat*_ (blue) for each cycle.

**Figure 9:**
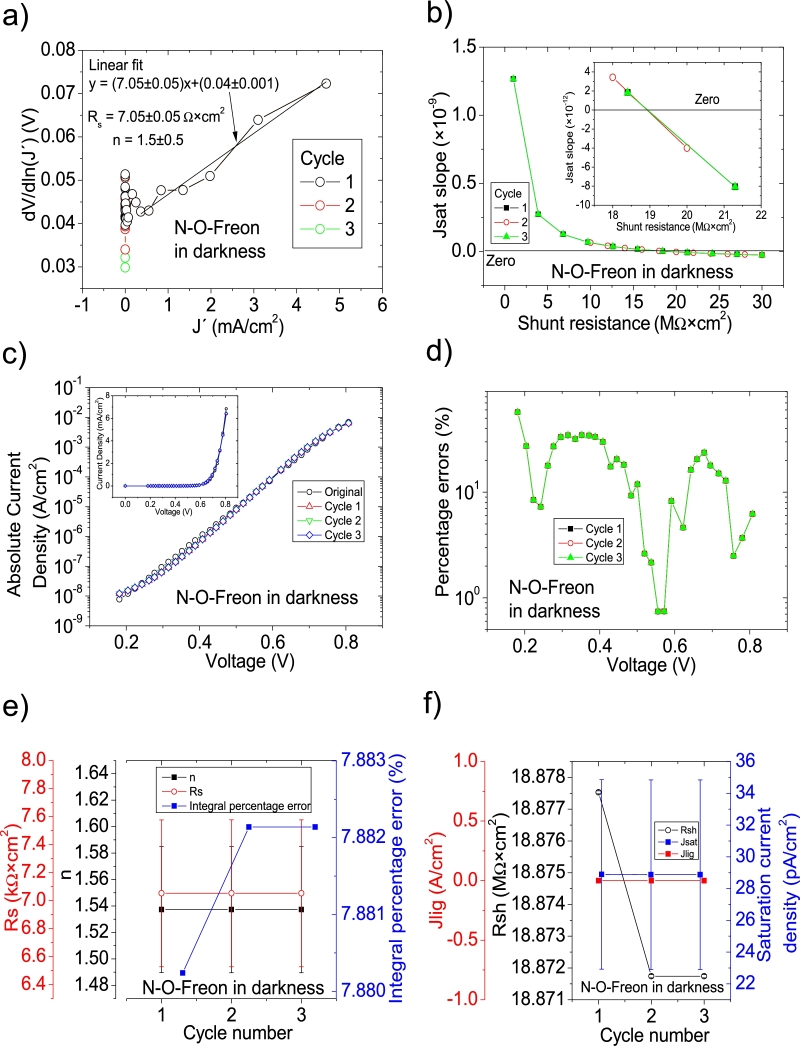
Three cycles application of program CycleB to the *JV* curve shown in Fig. 8.a) in [21] for the N-O-Freon curve in darkness, and the respective cycles steps shown as (a) linear fit of $\frac{\partial V}{\partial lnJ^{\prime }}=nkT+R_{s}\left(J^{\prime }\right)$ vs. *J*′, (b) plot of *m*_*sat*_*vs. R*_*sh*_ to obtain a root for *R*_*sh*_. (c) Logarithm plot of absolute *J* vs *V* of the original *JV* curve (in black) and for each resimulations done with the deduced solar cell. The same data are plot in the inset as *JV*. (d) Percentage errors between the original *JV* curve and each resimulation shown in (c). (e) Deduced *R*_*s*_ (red), *n* (black) and integral percentage errors (blue) for each cycle. For clarity purposes, the error bars were removed. (f) Deduced *R*_*sh*_ (black), *J*_*lig*_ (red) and *J*_*sat*_ (blue) for each cycle.

CycleA and CycleB were used to obtain the proper solar cell parameters, using the programs CycleAmanual and CycleB described in [5]. In the case of Ar and Ar-Freon using CycleB, it was impossible to find a root for $R_{sh}$ as the plot of $m_{Isat}$ *vs.* $R_{sh}$ was always positive (not shown here). Then, in these cases, CycleAmanual was used and they are shown in Fig. 6.abcdef) and Fig. 7.abcdef). Also, in these two cases, to obtain reasonable linear fit of $\frac{\partial V}{\partial lnJ^{\prime }}$ *vs.* $J^{\prime }$ it was necessary to do it only in the [0 V, 0.5 V] and [0 V, 0.8 V] for Ar in darkness and Ar-Freon darkness, respectively. Seven cycles were necessary in these cases to obtain reasonable convergence in all the solar cell parameters. Otherwise, the correlation was below 0.6. Then, in this article, the solar cell parameters extracted for these cases are only valid in these voltage ranges. In the cases of Ar-O-Freon and N-O-Freon, both programs were used, and it was CycleB the one that yielded the smallest percentage errors and integral percentage errors. Only three cycles were needed for these cases, to obtain convergence. They are shown in Fig. Figure 6, Figure 7, Figure 8, Figure 9. Results are summarized in Table 2.

**Table 2:** Solar cell parameters reported in [25], [26], [27] and deduced in this article. The superscript ^a^ refers to the sample name and value reported in [25], while the superscript ^b^ refers to the sample name and value reported in [26]. The superscript ^c^ refers to the sample name and value reported in [27]. The superscript ^d^ are the results obtained in this article, after applying the iterative cycles. In the very particular case of the superscript ^e^, they are the values reported in ^d^ for the Ar and Ar-Freon in darkness, after refinement. All values reported in ^d^, are after convergence of the integral percentage error, except for the cases Ar, Ar-Freon and Ar-O-Freon under illumination. In these cases, the values reported are for the first cycle. Further explanation can be found in the text.

Sample	$R_{sh}$ ($\Omega \times {\text{cm}}^{2}$)	$R_{s}$ ($\Omega \times {\text{cm}}^{2}$)	*n*	$J_{\text{sat}}$ (A/cm^2^)	$J_{\text{lig}}$ (mA/cm^2^)
In darkness					
	^a,b,c^10^7^	^a,b,c^ 750	^a,b,c^ 2.2	^a,b,c^ 2 × 10^−8^	0
^a^A, ^b,c^Ar	^d^ (1 ± 2) × 10^6^	^d^ (1.5 ± 0.3) × 10^3^	^d^ 2.3 ± 0.2	^d^ (3.61 ± 0.01) × 10^−8^	0
	^e^ 9 × 10^7^	^e^ 3.7 × 10^3^	^e^ 1.51	^e^ 1.11 × 10^−8^	
^a^ B, ^b^ArF, ^c^Ar-Freon	^a,b,c^10^7^	^a,b,c^ 50	^a,b,c^ 1.9	^a,b,c^ 3 × 10^−7^	0
	^d^ (6 ± 3) × 10^8^	^d^ 34 ± 3	^d^ 2.8 ± 0.1	^d^ (1.597 ± 0.001) × 10^−7^	0
	^e^ 6 × 10^7^	^e^ 43	^e^ 1.9	^e^ 7 × 10^−9^	
^a^ C, ^b^ArOF	^a^ 6 × 10^4^,	^a,b,c^ 9.2	^a,b,c^ 1.8	^a,b,c^ 1.5 × 10^−10^	0
^c^Ar-O-Freon	^b,c^ 6 × 10^6^				
	^d^ (7 ± 0.000005) × 10^6^	^d^ 13 ± 2	^d^ 1.8 ± 0.1	^d^ (3 ± 0.5) × 10^−10^	
^c^ N-O-Freon	^c^ 10^8^	^c^2.5	^c^ 1.78	^c^ 1.4 × 10^−10^	0
	^d^ (1.887174 ± 0.00005) × 10^7^	^d^ 7 ± 1	^d^ 1.54 ± 0.05	^d^ (2.9 ± 0.6) × 10^−11^	0
Under illumination					
^a^A, ^b,c^Ar	^a,b,c^ 210	^a,b^13			^a,b^ 16.6
	^d^ (14 ± 5) × 10^4^	^d^ 9.5 ± 0.6	^d^ 4.7 ± 0.2	^d^ (1.32 ± 0.05) × 10^−6^	^d^ 15.6
^a^ B, ^b^ArF, ^c^Ar-Freon	^a,b,c^200	^a,b^ 9			^a,b^ 22.1
	^d^ 5309.7 ± 0.5	^d^ 7.1 ± 0.5	^d^ 3.9 ± 0.3	^d^ (3.5 ± 0.1) × 10^−5^	^d^ 21.1
^a^ C, ^b^ ArOF,	^a,b,c^ 740	^a,b^ 5			^a,b^ 25.5
^c^Ar-O-Freon	^d^ 1865.5 ± 0.5	^d^ 3.5 ± 0.4	^d^ 6.6 ± 0.3	^d^ (3 ± 0.06) × 10^−4^	^d^ 25.5
^c^ N-O-Freon	^c^ 2000				
	^d^ 697.4 ± 0.5	^d^ 6.7 ± 0.3	^d^ 1.8 ± 0.2	^d^ (1.3 ± 0.2) × 10^−9^	^d^ 22.0

**Figure 10:**
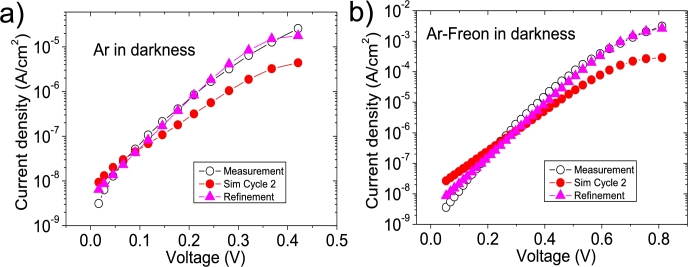
(a) Original Ar measurement (open black circle), simulation (red circles) using Cycle 2 (smallest errors obtained) values shown in Fig. 6.abcdef), and simulation (purple triangles) using the refinement of these last values (*R*_*sh*_ = 9 × 10^7^ Ω × cm^2^, *R*_*s*_ = 3.7 × 10^3^ Ω × cm^2^, *n* = 1.51, $J_{sat}=1.11\times 10^{-8}\;\frac{\text{A}}{{\text{cm}}^{2}}$), (b) original Ar-Freon measurement (open black circle), simulation (red circles) using Cycle 2 (smallest errors obtained) values shown in Fig. 7.abcdef), and simulation (purple triangles) using the refinement of these last values (*R*_*sh*_ = 6 × 10^7^ Ω × cm^2^, *R*_*s*_ = 43 Ω × cm^2^, *n* = 1.9, $J_{sat}=7\times 10^{-9}\;\frac{\text{A}}{{\text{cm}}^{2}}$).

In the case of the deduced solar cell parameters for the Ar and Ar-Freon cases, errors were around 10% or larger. Then, using trial and error, the solar cell parameters were improved, obtaining for the Ar (Ar-Freon) case values of $R_{sh}=9\times 10^{7}\;\Omega \times {\text{cm}}^{2}\;\left(6\times 10^{7}\;\Omega \times {\text{cm}}^{2}\right)$, $R_{s}=3.7\times 10^{3}\;\Omega \times {\text{cm}}^{2}\;\left(43\;\Omega \times {\text{cm}}^{2}\right)$, $n=1.51\;(1.9),\;J_{sat}=1.11\times 10^{-8}\;\frac{\text{A}}{{\text{cm}}^{2}}$ ($7\times 10^{-9}\;\frac{\text{A}}{{\text{cm}}^{2}}$). They are shown in Fig. 10.ab), and summarized with the superscript ^e^ in Table 3. This fact reveals, that in some cases, the iterative cycles might not yield accurate enough solar cell parameters, but, nevertheless, they are good approximations, that can be enhanced using trial and error.

**Table 3:** Solar cell parameters for the solar cells studied in [28]. The superscript ^a^ refers to the sample name and value reported in [28], while the superscript ^b^ refers to the values deduced in this study, considering the *JV* without the roll-over region, while superscript ^c^ considers the roll-over region.

Measurement	*R*_*sh*_ (Ω × cm^2^)	*R*_*s*_ (Ω × cm^2^)	*n*	$J_{\text{sat}}$ (mA/cm^2^)	$J_{\text{lig}}$ (mA/ cm^2^)
	^a^ 1300	^a^ 6.8			^a^ 27.85
15 nm TiO_2_	^b^ 356.633 ± 0.005	^b^ 5 ± 0.7	^b^ 2.5 ± 0.2	^b^ (2.3 ± 0.4) × 10^−4^	^b^ 28.3
	^c^ 508.398 ± 0.005	^c^ 7.8 ± 0.5	^c^ 2 ± 0.2	^c^ (9.86 ± 0.03) × 10^−3^	^c^ 28.33
	^a^ 1200	11.6			24.62
30 nm TiO_2_	^b^ 1297.193 ± 0.005	^b^ 7 ± 2	^b^ 3.4 ± 0.4	^b^ (1.7 ± 0.2) × 10^−2^	^b^ 24.63
	^c^ 256.92 ± 0.005	^c^ 11 ± 2	^c^ 2.3 ± 0.3	^c^ (1.6 ± 0.7) × 10^−4^	^c^ 25.48

### 3.2 Measurements under illumination

**Figure 11:**
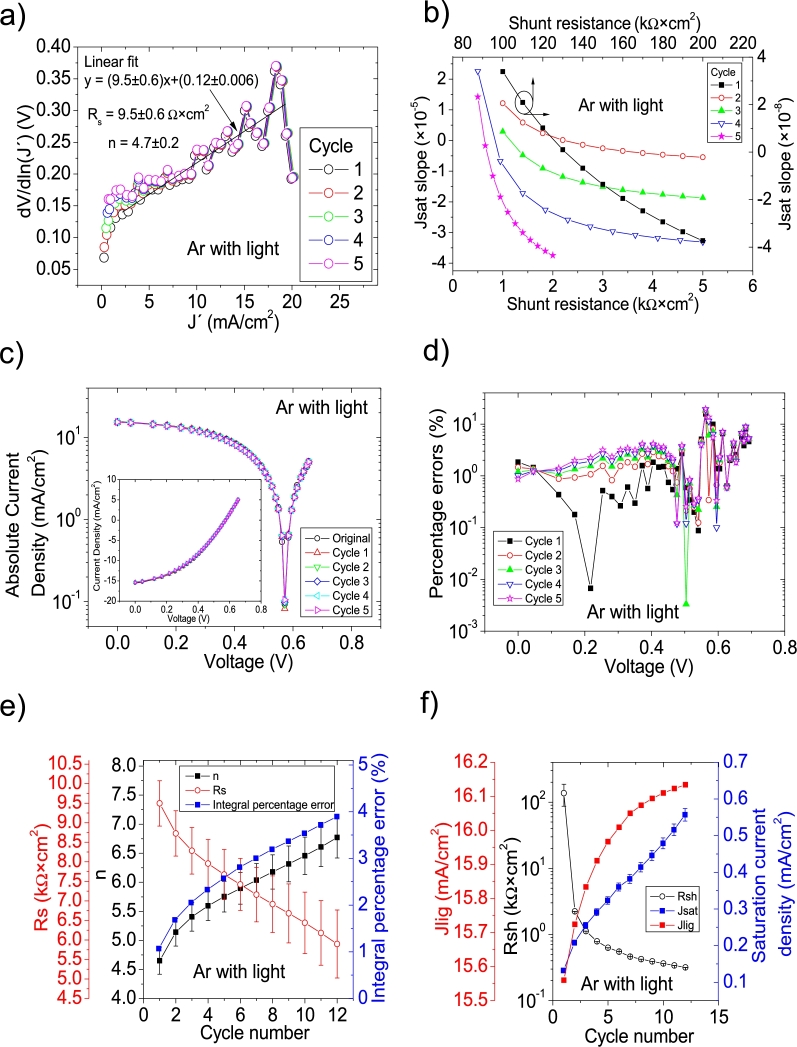
Twelve cycles application of program CycleB to the *JV* curve shown in Fig. 8.b) in [21] for the Ar curve under illumination, and the respective cycles steps shown as (a) linear fit of $\frac{\partial V}{\partial lnJ^{\prime }}=nkT+R_{s}\left(J^{\prime }\right)$ vs. *J*′, (b) plot of *m*_*sat*_*vs. R*_*sh*_ to obtain a root for *R*_*sh*_, for the first five cycles. (c) Logarithm plot of absolute *J* vs *V* of the original *JV* curve (in black) and for each resimulations done with the deduced solar cell. The same data are plot in the inset as *JV*. (d) Percentage errors between the original *JV* curve and each resimulation shown in (c). (e) Deduced *R*_*s*_ (red), *n* (black) and integral percentage errors (blue) for each cycle. For clarity purposes, the error bars were removed. (f) Deduced *R*_*sh*_ (black), *J*_*lig*_ (red) and *J*_*sat*_ (blue) for each cycle.

**Figure 12:**
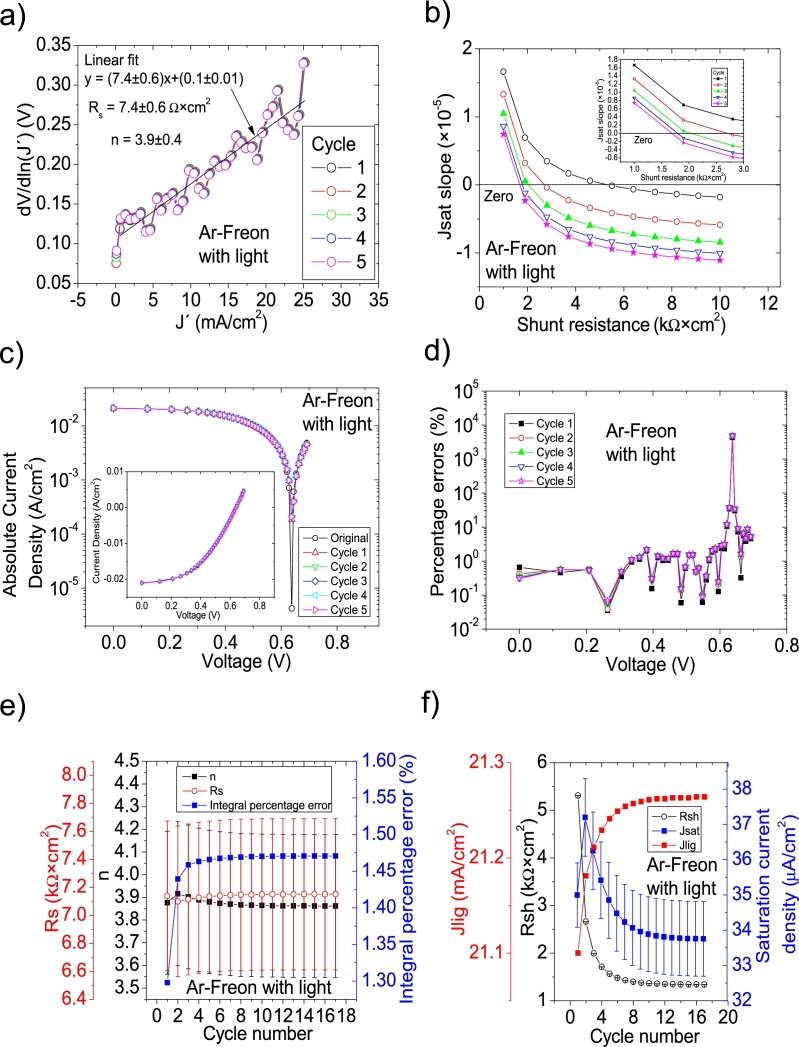
Seventeen cycles application of program CycleB to the *JV* curve shown in Fig. 8.b) in [21] for the Ar-Freon curve under illumination, and the respective cycles steps shown as (a) linear fit of $\frac{\partial V}{\partial lnJ^{\prime }}=nkT+R_{s}\left(J^{\prime }\right)$ vs. *J*′, (b) plot of *m*_*sat*_*vs. R*_*sh*_ to obtain a root for *R*_*sh*_, for the first five cycles. (c) Logarithm plot of absolute *J* vs *V* of the original *JV* curve (in black) and for each resimulations done with the deduced solar cell. The same data are plot in the inset as *JV*. (d) Percentage errors between the original *JV* curve and each resimulation shown in (c). (e) Deduced *R*_*s*_ (red), *n* (black) and integral percentage errors (blue) for each cycle. For clarity purposes, the error bars were removed. (f) Deduced *R*_*sh*_ (black), *J*_*lig*_ (red) and *J*_*sat*_ (blue) for each cycle.

**Figure 13:**
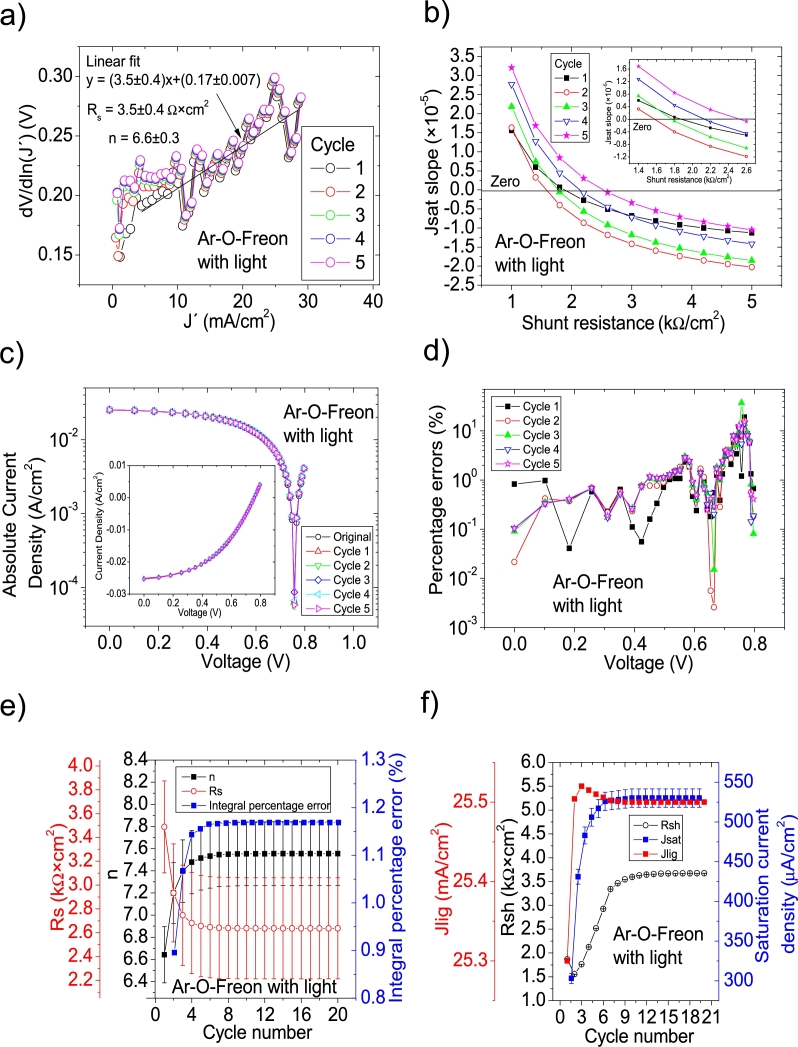
Twenty cycles application of program CycleB to the *JV* curve shown in Fig. 8.b) in [21] for the Ar-O-Freon curve under illumination, and the respective cycles steps shown as (a) linear fit of $\frac{\partial V}{\partial lnJ^{\prime }}=nkT+R_{s}\left(J^{\prime }\right)$ vs. *J*′, (b) plot of *m*_*sat*_*vs. R*_*sh*_ to obtain a root for *R*_*sh*_, for the first five cycles. (c) Logarithm plot of absolute *J* vs *V* of the original *JV* curve (in black) and for each resimulations done with the deduced solar cell. The same data are plot in the inset as *JV*. (d) Percentage errors between the original *JV* curve and each resimulation shown in (c). (e) Deduced *R*_*s*_ (red), *n* (black) and integral percentage errors (blue) for each cycle. For clarity purposes, the error bars were removed. (f) Deduced *R*_*sh*_ (black), *J*_*lig*_ (red) and *J*_*sat*_ (blue) for each cycle.

**Figure 14:**
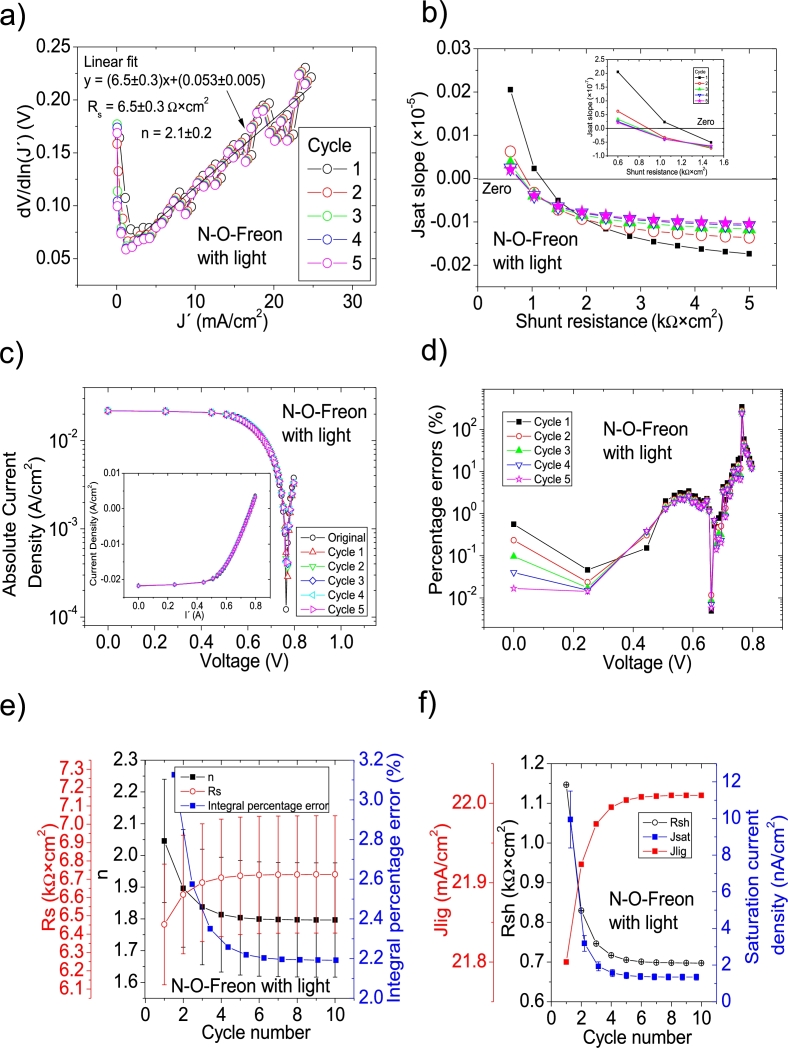
Ten cycles application of program CycleB to the *JV* curve shown in Fig. 8.b) in [21] for the N-O-Freon curve under illumination, and the respective cycles steps shown as (a) linear fit of $\frac{\partial V}{\partial lnJ^{\prime }}=nkT+R_{s}\left(J^{\prime }\right)$ vs. *J*′, (b) plot of *m*_*sat*_*vs. R*_*sh*_ to obtain a root for *R*_*sh*_, for the first five cycles. (c) Logarithm plot of absolute *J* vs *V* of the original *JV* curve (in black) and for each resimulations done with the deduced solar cell. The same data are plot in the inset as *JV*. (d) Percentage errors between the original *JV* curve and each resimulation shown in (c). (e) Deduced *R*_*s*_ (red), *n* (black) and integral percentage errors (blue) for each cycle. For clarity purposes, the error bars were removed. (f) Deduced *R*_*sh*_ (black), *J*_*lig*_ (red) and *J*_*sat*_ (blue) for each cycle.

As it is discussed in Section 3 in [21], not all the solar cell parameters were reported in this case (see Table 3 in [25], Table II in [26] and Table II in [27]), despite it is claimed in [25] that the Ortiz-Conde et al. [24] method was used. In fact, as it is discussed in Section 3 in [21], it was impossible that it was used as no *J* measured at 0 V was reported (see discussion in Section 3 in [21]). Then, it was impossible to simulate the *JV* curves, to confirm the proper solar cell parameter extraction. At the same, in Section 3 in [21], the Ortiz-Conde et al. method was used in the *JV* curves under illumination, always obtaining negative values for $R_{s}$ and *n* (see Table 5 in [21]). Then it is unclear how the solar cell parameters reported in [25], [26], [27] were deduced. These are obtained in this Subsection, using CycleB in the *JV* curves shown in Fig. 8.b) in [21]. Also, CycleA was used (not shown here), but it was CycleB which yielded the smallest percentage errors. The application of CycleB is shown in Fig. Figure 11, Figure 12, Figure 13, Figure 14. For the case of N-O-Freon, the integral percentage error decreased, as the percentage errors as the cycle reached the value of ten (it is the solar cell parameters deduced in this last cycle that are reported in Table 2). On the contrary, the integral percentage error increased for Ar, Ar-Freon and Ar-O-Freon cases, diverging for the Ar case, while converging for the other two cases. In all these three cases, the smallest percentage errors were obtained for Cycle 1, and they are those reported in Table 2.

The application of the iterative cycles reveals that the value of $J_{sat}$ in the case of the Ar-Freon sample in darkness reported in Table 2 in [25] is two orders of magnitude smaller than the correct value. Something similar happens with the value of $R_{sh}$ for the Ar-O-Freon in darkness reported in Table 2 in [25], which is two orders of magnitude smaller than the correct value. Other discrepancies for $R_{s}$ and *n* can be found for the cases measured in darkness.

In the case of measurements done under illumination, one or two orders of magnitude discrepancies can be observed for $R_{sh}$, while some tens percentage errors can be seen regarding *n*.

The results exposed in this Section show the suitability of the iterative cycles to obtain the solar cell parameters, despite in some cases, the values are not as accurate, but they can be easily enhanced using trial and error procedure. Also, the results given in this section suggest that conclusions exposed in [25], [26], [27] should be revised.

## 4 IV measurements done with $P_{V}\geq 50\;\frac{measured\;points}{V}$ and $P_{V}<100\;\frac{measured\;points}{V}$

**Figure 15:**
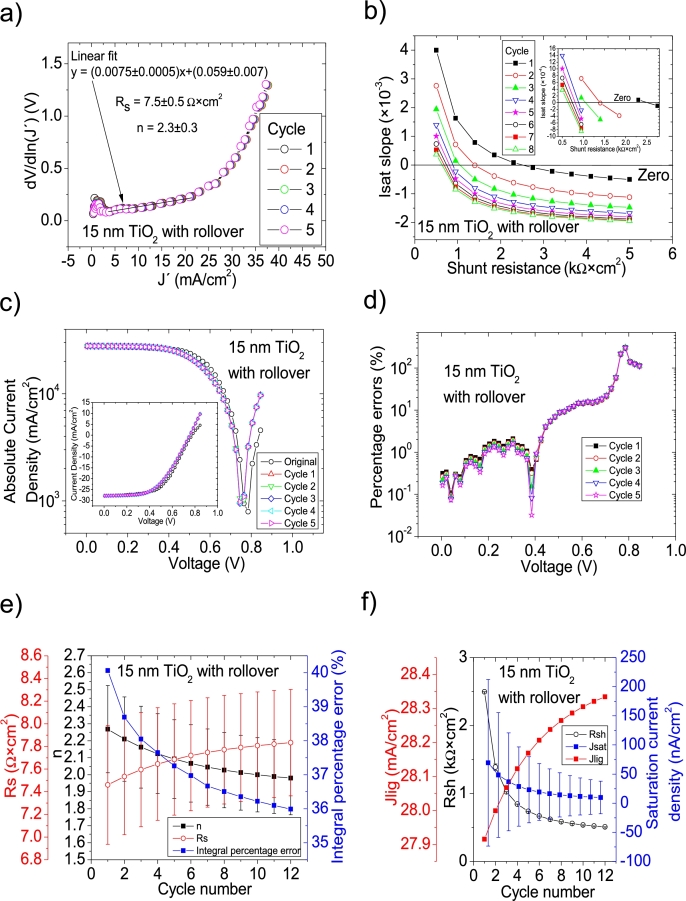
Twelve cycles application of program CycleB to the *JV* curve shown in Fig. 12 in [21] for the 15 nm TiO2 (including the roll over) curve under illumination, and the respective cycles steps shown as (a) linear fit of $\frac{\partial V}{\partial lnJ^{\prime }}=nkT+R_{s}\left(J^{\prime }\right)$ vs. *J*′, for the first five cycles, (b) plot of *m*_*sat*_*vs. R*_*sh*_ to obtain a root for *R*_*sh*_, for the first eight cycles. (c) Logarithm plot of absolute *J* vs *V* of the original *JV* curve (in black) and for each resimulations done with the deduced solar cell, for the first five cycles. The same data are plot in the inset as *JV*. (d) Percentage errors between the original *JV* curve and each resimulation shown in (c), for the first five cycles. (e) Deduced *R*_*s*_ (red), *n* (black) and integral percentage errors (blue) for each cycle. (f) Deduced *R*_*sh*_ (black), *J*_*lig*_ (red) and *J*_*sat*_ (blue) for each cycle.

**Figure 16:**
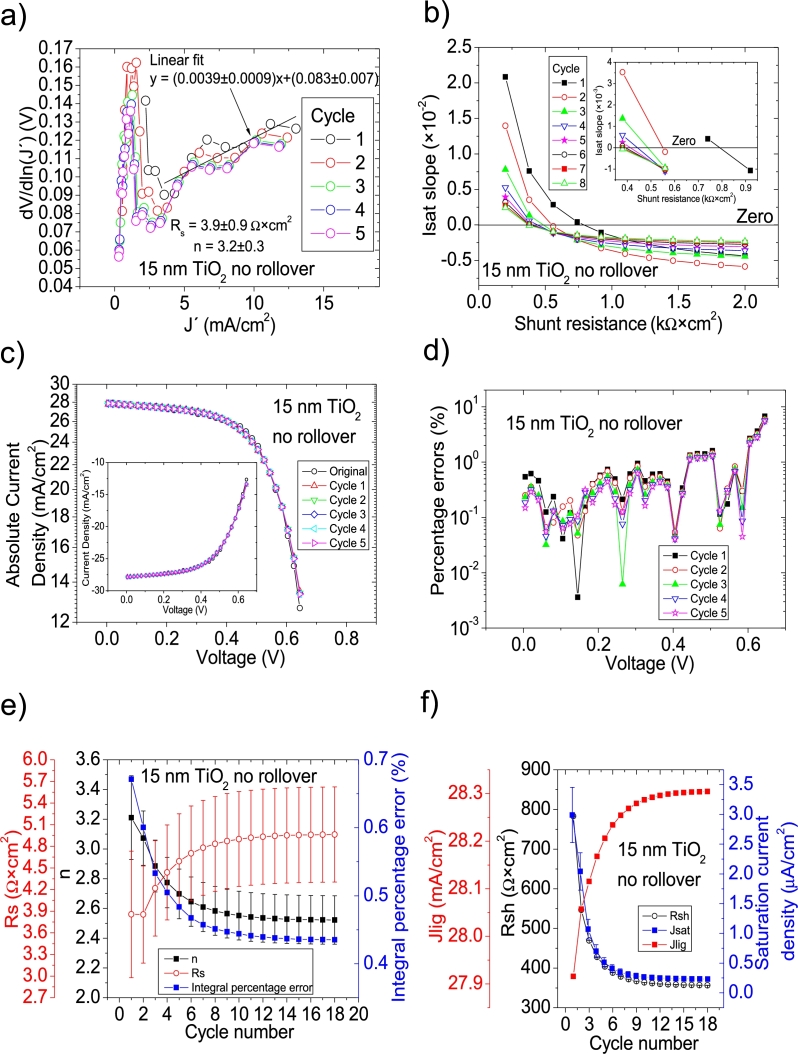
Eighteen cycles application of program CycleB to the *JV* curve shown in Fig. 12 in [21] for the 15 nm TiO2 (excluding roll over) curve under illumination, and the respective cycles steps shown as (a) linear fit of $\frac{\partial V}{\partial lnJ^{\prime }}=nkT+R_{s}\left(J^{\prime }\right)$ vs. *J*′, for the first five cycles, (b) plot of *m*_*sat*_*vs. R*_*sh*_ to obtain a root for *R*_*sh*_, for the first eight cycles. (c) Logarithm plot of absolute *J* vs *V* of the original *JV* curve (in black) and for each resimulations done with the deduced solar cell, for the first five cycles. The same data are plot in the inset as *JV*. (d) Percentage errors between the original *JV* curve and each resimulation shown in (c), for the first five cycles. (e) Deduced *R*_*s*_ (red), *n* (black) and integral percentage errors (blue) for each cycle. (f) Deduced *R*_*sh*_ (black), *J*_*lig*_ (red) and *J*_*sat*_ (blue) for each cycle.

**Figure 17:**
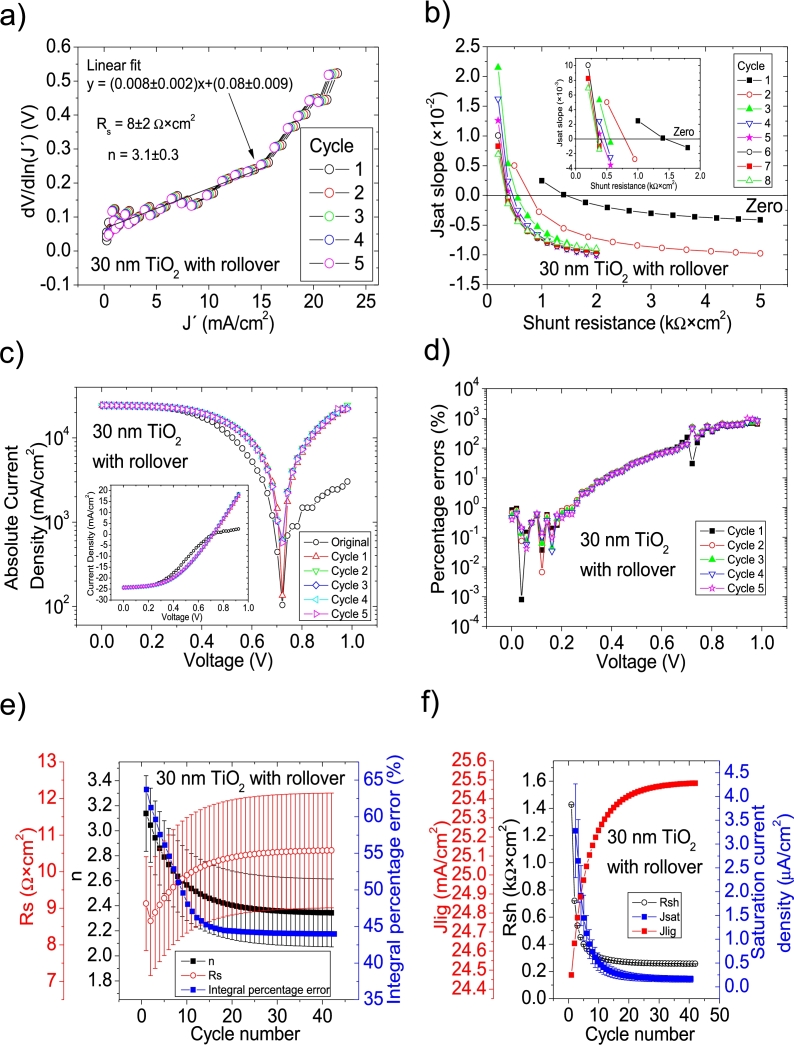
Forty cycles application of program CycleB to the *JV* curve shown in Fig. 12 in [21] for the 30 nm TiO2 (including the roll over) curve under illumination, and the respective cycles steps shown as (a) linear fit of $\frac{\partial V}{\partial lnJ^{\prime }}=nkT+R_{s}\left(J^{\prime }\right)$ vs. *J*′, for the first five cycles, (b) plot of *m*_*sat*_*vs. R*_*sh*_ to obtain a root for *R*_*sh*_, for the first eight cycles. (c) Logarithm plot of absolute *J* vs *V* of the original *JV* curve (in black) and for each resimulations done with the deduced solar cell, for the first five cycles. The same data are plot in the inset as *JV*. (d) Percentage errors between the original *JV* curve and each resimulation shown in (c), for the first five cycles. (e) Deduced *R*_*s*_ (red), *n* (black) and integral percentage errors (blue) for each cycle. (f) Deduced *R*_*sh*_ (black), *J*_*lig*_ (red) and *J*_*sat*_ (blue) for each cycle.

**Figure 18:**
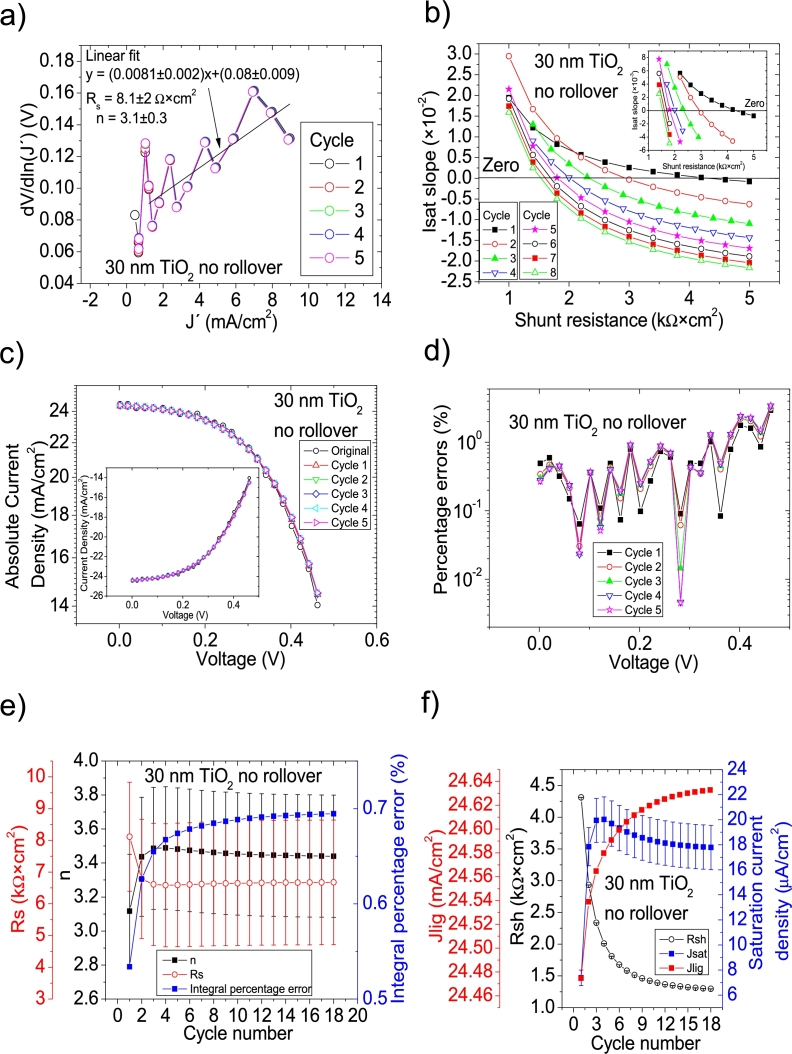
Eighteen cycles application of program CycleB to the *JV* curve shown in Fig. 12 in [21] for the 30 nm TiO2 (excluding roll over) curve under illumination, and the respective cycles steps shown as (a) linear fit of $\frac{\partial V}{\partial lnJ^{\prime }}=nkT+R_{s}\left(J^{\prime }\right)$ vs. *J*′, for the first five cycles, (b) plot of *m*_*sat*_*vs. R*_*sh*_ to obtain a root for *R*_*sh*_, for the first eight cycles. (c) Logarithm plot of absolute *J* vs *V* of the original *JV* curve (in black) and for each resimulations done with the deduced solar cell, for the first five cycles. The same data are plot in the inset as *JV*. (d) Percentage errors between the original *JV* curve and each resimulation shown in (c), for the first five cycles. (e) Deduced *R*_*s*_ (red), *n* (black) and integral percentage errors (blue) for each cycle. (f) Deduced *R*_*sh*_ (black), *J*_*lig*_ (red) and *J*_*sat*_ (blue) for each cycle.

Hernández-Rodríguez et al. [28] mentioned they used the Ortiz-Conde et al. [24] method to determine the solar cell parameters of their CdS/CdTe solar cells, and reported only $R_{s}$ and $R_{sh}$ in their Table 2 in [28], making impossible to confirm their accuracy, simulating the curves. Their illuminated *JV* measurements had $P_{V}=50\;\frac{measured\;points}{V}$ (see Fig. 4.a) in [28]. The Ortiz-Conde et al. method [24] was attempted on their *JV* curves in Section 4 in [21] and, as it is discussed there, in every case, both including and excluding the roll-over effect, $R_{s}$ and/or *n* were unrealistically negative (see Table 6 in [21]). Then, it is unclear how their reported $R_{s}$ and $R_{sh}$ in their Table 2 in [28] were obtained and if they are accurate. For simplicity purposes for the reader, the same labelling as in [28] is used in this article. CycleA and CycleB were used in their 15 nm TiO_2_, 30 nm TiO_2_, and 60 nm TiO_2_ shown in Fig. 12 in [21], to obtain the solar cell parameters. In the case of the 60 nm TiO_2_, it was impossible to apply both cycles, as the *JV* curve does not show any rectification nature (see Fig. 12 in [21]). This shows that a minimum rectifying nature is necessary in the *IV* or *JV* curves, for the iterative cycles to work. CycleA and CycleB were tried on the *JV* measurements for the 15 nm TiO_2_ and 30 nm TiO_2_, both in the [0 V, 1 V] (including the roll-over effect) and for voltage ranges of [0 V, 0.645 V] and [0 V, 0.4 V], i.e., when the roll-over effect is not present, respectively. It was again CycleB the one that yielded the smallest percentage errors, and they are shown in Fig. Figure 15, Figure 16, Figure 17, Figure 18. In the case when the roll-over was considered, the linear fit of $\frac{\partial V}{\partial lnJ^{\prime }}=nkT+R_{s}\left(J^{\prime }\right)$ vs. $J^{\prime }$ was done in the voltage range where the roll-over was not present. Results are summarized in Table 3.

Interestingly, $R_{sh}$ is similar to their reported value for the 30 nm TiO2 case with no rollover, however, the correct $R_{sh}$ is between 2 and 4 times smaller than their reported value for 15 nm TiO2 case. Regarding $R_{s}$, they are in reasonable agreement with their reported value, especially for the case 30 nm TiO2 case with rollover. The results exposed in this Section show that CycleB can be applied even in the case when rollover is present, provided that the linear fit of $\frac{\partial V}{\partial lnJ^{\prime }}$ *vs.* $J^{\prime }$ is done in the voltage region before the roll-over happens. Nevertheless, the results exposed here show that the results exposed in [28] should be revised, regarding their $R_{s}$ and $R_{sh}$.

**Figure 19:**
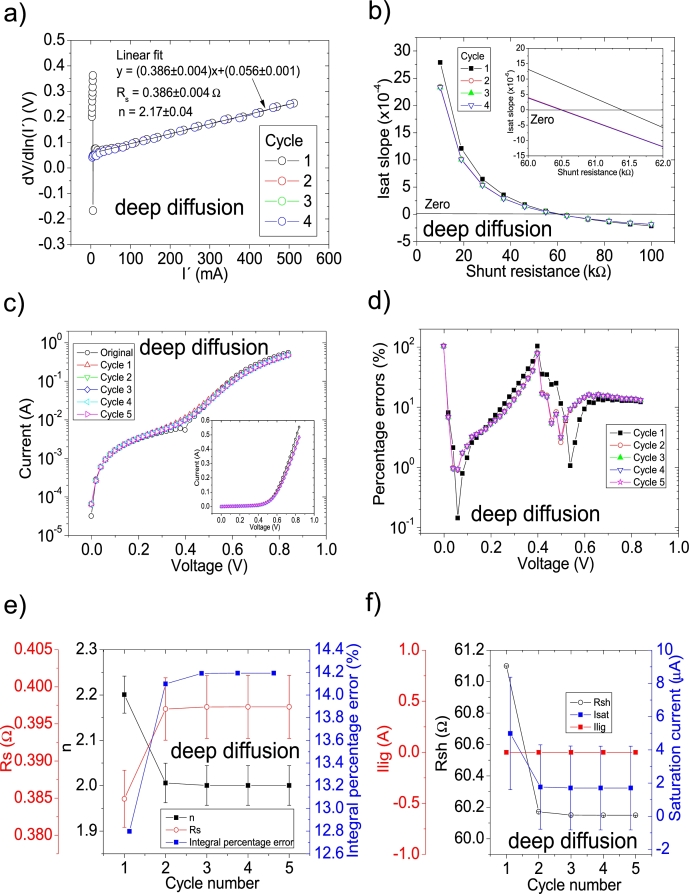
Five cycles application of program CycleB to the *JV* curve shown in Fig. 15.a) in [21] for the deep diffusion curve in darkness, and the respective cycles steps shown as (a) linear fit of $\frac{\partial V}{\partial lnJ^{\prime }}=nkT+R_{s}\left(J^{\prime }\right)$ vs. *J*′, for the first four cycles, (b) plot of *m*_*sat*_*vs. R*_*sh*_ to obtain a root for *R*_*sh*_, for the first four cycles. (c) Logarithm plot of absolute *J* vs *V* of the original *JV* curve (in black) and for each resimulations done with the deduced solar cell. The same data are plot in the inset as *JV*. (d) Percentage errors between the original *JV* curve and each resimulation shown in (c). (e) Deduced *R*_*s*_ (red), *n* (black) and integral percentage errors (blue) for each cycle. (f) Deduced *R*_*sh*_ (black), *J*_*lig*_ (red) and *J*_*sat*_ (blue) for each cycle.

**Figure 20:**
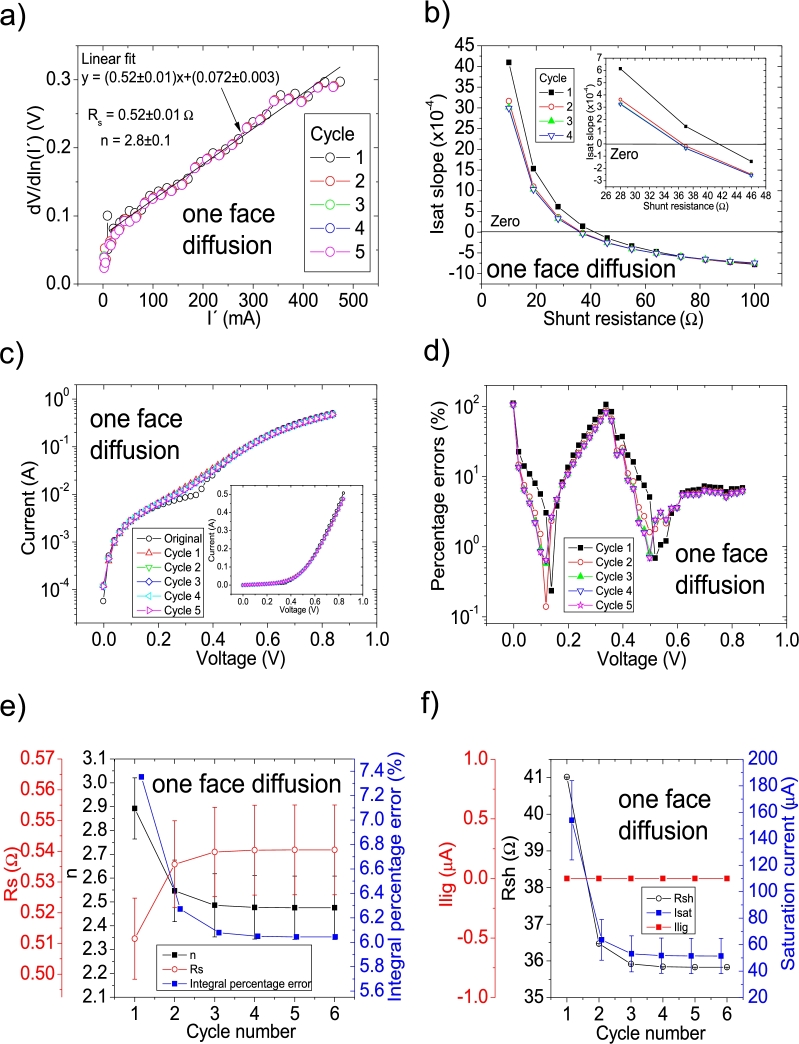
Six cycles application of program CycleB to the *JV* curve shown in Fig. 15.a) in [21] for the one face diffusion curve in darkness, and the respective cycles steps shown as (a) linear fit of $\frac{\partial V}{\partial lnJ^{\prime }}=nkT+R_{s}\left(J^{\prime }\right)$ vs. *J*′, for the first five cycles, (b) plot of *m*_*sat*_*vs. R*_*sh*_ to obtain a root for *R*_*sh*_, for the first four cycles. (c) Logarithm plot of absolute *J* vs *V* of the original *JV* curve (in black) and for each resimulations done with the deduced solar cell, for the first five cycles. The same data are plot in the inset as *JV*. (d) Percentage errors between the original *JV* curve and each resimulation shown in (c), for the first five cycles. (e) Deduced *R*_*s*_ (red), *n* (black) and integral percentage errors (blue) for each cycle. (f) Deduced *R*_*sh*_ (black), *J*_*lig*_ (red) and *J*_*sat*_ (blue) for each cycle.

**Figure 21:**
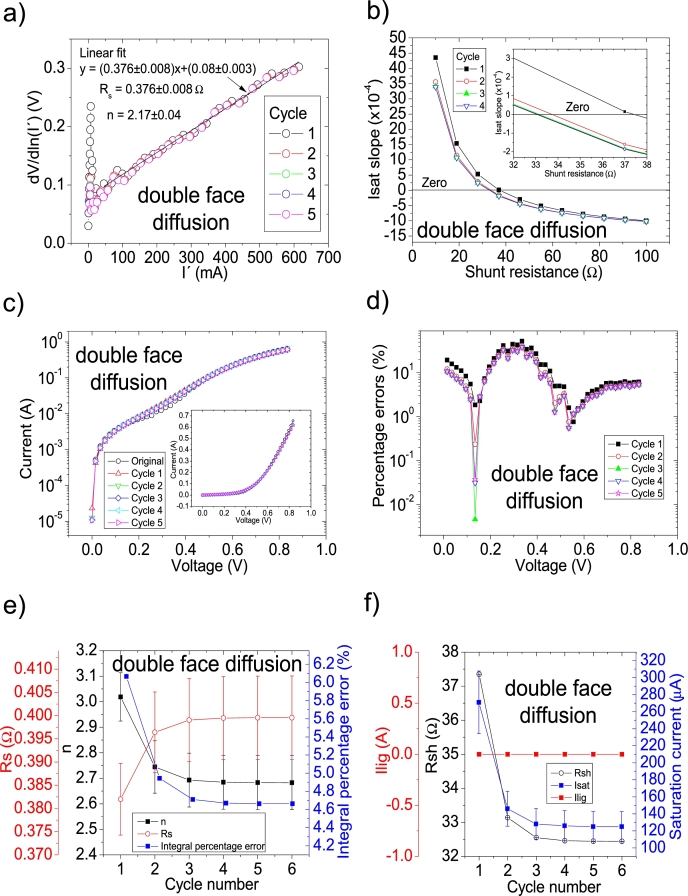
Six cycles application of program CycleB to the *JV* curve shown in Fig. 15.a) in [21] for the double face diffusion curve in darkness, and the respective cycles steps shown as (a) linear fit of $\frac{\partial V}{\partial lnJ^{\prime }}=nkT+R_{s}\left(J^{\prime }\right)$ vs. *J*′, for the first five cycles, (b) plot of *m*_*sat*_*vs. R*_*sh*_ to obtain a root for *R*_*sh*_, for the first four cycles. (c) Logarithm plot of absolute *J* vs *V* of the original *JV* curve (in black) and for each resimulations done with the deduced solar cell, for the first five cycles. The same data are plot in the inset as *JV*. (d) Percentage errors between the original *JV* curve and each resimulation shown in (c), for the first five cycles. (e) Deduced *R*_*s*_ (red), *n* (black) and integral percentage errors (blue) for each cycle. (f) Deduced *R*_*sh*_ (black), *J*_*lig*_ (red) and *J*_*sat*_ (blue) for each cycle.

In another study, the impact of metal impurities and illumination on the solar cell parameters prepared on mono-silicon wafers was reported by Li et al. [29]. The same labelling as in [29] is used in this article. They reported their deduced solar cell parameters (Table 1 in [29]), using the Ortiz-Conde et al. [24] procedure. Their $P_{V}=52\;\frac{measured\;points}{V}$ in this case. As it is discussed in Section 4 in [21], the *IV* curves are not simulated using their deduced solar cell parameters, revealing an incorrect solar cell parameter deduction. CycleA and CycleB were used to obtain the correct solar cell parameters. Again, it was CycleB which yielded the smallest percentage errors, and its application is shown in Fig. Figure 19, Figure 20, Figure 21. The same labelling as in [29] is used.

The application of the iterative cycles reveals that the value of $R_{sh}$ is overestimated in the results in [29] and it is smaller. Also, discrepancies are found for $R_{s}$ and *n* (see Table 4).

**Table 4:** Solar cell parameters for the solar cells studied in [29]. The superscript ^a^ are the data reported for samples Team 1 in Table 1 in [29]. The superscript ^b^ are the results obtained in this article.

Measurement	*R*_*sh*_ (Ω)	*R*_*s*_ (Ω)	*n*	$I_{\text{sat}}$ (A)
^a^ one face diffusion	^a^ 129.33	^a^ 0.58	^a^ 3.59	^a^ 2.2 × 10^−4^
	^b^ 35.82789 ± 0.00005	^b^ 0.54 ± 0.01	^b^ 2.5 ± 0.1	^b^ (5.1 ± 0.1) × 10^−5^
^a^ double face diffusion	^a^ 186.26	^a^ 0.22	^a^ 2.03	^a^ 1.5 × 10^−4^
	^b^ 32.44232 ± 0.00005	^b^ 0.4 ± 0.009	^b^ 2.7 ± 0.1	^b^ (1.2 ± 0.2) × 10^−4^
^a^ deep diffusion	^a^ 99.38	^a^ 0.069	^a^ 2.79	^a^ 2.3 × 10^−6^
	^b^ 60.14977 ± 0.00005	^b^ 0.4 ± 0.004	^b^ 2 ± 0.04	^b^ (2 ± 3) × 10^−6^

In the case of measurements done under illumination, one or two orders of magnitude discrepancies can be observed for $R_{sh}$, while some tens percentage errors can be seen regarding *n*.

The results exposed in this Section show the suitability of the iterative cycles to obtain the solar cell parameters, and at the same time, the conclusions given in [29] should be revised.

## 5 Conclusions

The cycles CycleA and CycleB have been applied to different *IV* and *JV* curves reported in the literature. In general, CycleB is the one that yields the most accurate solar cell parameters. However, situations were found when it was not possible to apply CycleB, as the plot of $m_{sat}$ *vs.* $R_{sh}$ never changed sign and then, it was impossible to obtain a root for $R_{sh}$. In these cases, CycleA become handy and was used to determine the solar cell parameters.

It was shown that CycleB could be used in situations when $P_{V}$ is as small as $1.6\;\frac{measured\;points}{V}$, provided the voltage range is around [0 V, 20 V]. Also, it was discovered that a condition on $\frac{R_{s}}{R_{sh}}$ exists for the cycles to be appliable, as a value of $\frac{R_{s}}{R_{sh}}=0.043$ causes the plot of $\frac{\partial V}{\partial lnI^{\prime }}=nkT+R_{s}\left(I^{\prime }\right)$ vs. $I^{\prime }$, to not be linear for large values of $I^{\prime }$, hindering the determination of $R_{s}$ and *n*. Also, in the case of CycleB, it caused the plot of $m_{sat}$ *vs.* $R_{sh}$ to be always positive and increasing, making it impossible to obtain a root for $R_{sh}$ while in the case of CycleA, $R_{sh}$ was found to be always unrealistically negative. The condition on $\frac{R_{s}}{R_{sh}}$ is currently being investigated and will be reported elsewhere.

In general, it was found that the percentage integral errors diminish and converge, as the number of cycles increases. However, in three cases it was found that it increased. In two of them it eventually converged, and in the last one it kept diverging. In these situations, the most accurate solar cell parameters are the one deduced in the first cycle.

In two other situations, the solar cell parameters extracted were not as accurate as one would like. Nevertheless, they are good approximations, and using trial and error, they were easily improved.

It was also shown that the proposed cycles can be used in situations where the roll-over effect is present. In these cases, the linear fit of $\frac{\partial V}{\partial lnI^{\prime }}=nkT+R_{s}\left(I^{\prime }\right)$ vs. $I^{\prime }$ should be done in the voltage range were the roll-over is not present. Good solar cell parameters were achieved in these cases.

It is worth mentioning the utility of CycleA and CycleB, to determine solar cell parameters such as *n*, and $I_{\mathrm{sat}}$, which are not so easily obtained using other methods (see discussion in [21], [22]). Also, a more accurate determination of $I_{\mathrm{lig}}$ is obtained, improving the usually used approximation of $I_{lig}≅I_{sc}$.

## Declarations

### Author contribution statement

Victor Tapio Rangel Kuoppa, Dr: Conceived and designed the experiments; Performed the experiments; Analyzed and interpreted the data; Contributed reagents, materials, analysis tools or data; Wrote the paper.

### Funding statement

Victor Tapio Rangel Kuoppa was supported by the 10.13039/100010897Newton Fund (RCUK-CONACyT 2016 FONCICYT/68).

### Data availability statement

Data will be made available on request.

### Declaration of interests statement

The authors declare no conflict of interest.

### Additional information

No additional information is available for this paper.
